# Effects of two kinds of marine algae polysaccharides and their oligosaccharides on lipid metabolism and gut microbiota in high-fat diet mice

**DOI:** 10.1128/spectrum.02832-24

**Published:** 2025-09-25

**Authors:** Zi-ang Yao, Ling Xu, Yu Liu, Hai-ge Wu

**Affiliations:** 1 College of Life Science, Dalian Minzu University, Dalian, Liaoning, China; 2 College of Life and Health, Dalian University, Dalian, Liaoning, China; Yangzhou University, Yangzhou, Jiangsu, China

**Keywords:** gut microbiota, lipid metabolism, sodium alginate, agar, oligosaccharides

## Abstract

Abnormal lipid metabolism is manifested in a variety of metabolic diseases. In recent years, studies have shown that the pathogenesis of these disorders is closely associated with the gut microbiota. Marine algae polysaccharides, such as sodium alginate and its oligosaccharides, can reduce blood lipids and improve lipid metabolism to reduce body weight. Polysaccharides are difficult to be absorbed by the human body due to their large molecular weight, which limits their application in the field of medicine and food. In this study, two kinds of marine algae polysaccharides and their oligosaccharides were used to investigate their effects on body weight, lipid metabolism, inflammatory factors, and intestinal health and microbiota in high-fat diet mice. The results showed that after feeding a high-fat diet, the mice’s body weight and serum lipids were increased. The formation of fat droplets was observed in the liver cells. Small intestinal microvilli were destroyed and showed obvious accumulation of epithelial cells. After feeding mice with sodium alginate, agar, and its oligosaccharides, the level of body weight and the pathological damage of the liver and intestine were relieved. Gut microbiota analysis showed that two kinds of polysaccharides and their oligosaccharides could improve the composition of gut microbiota at the phylum and genus level, increase the abundance of *Lactobacillus* and *Akkermansia*, and reduce the abundance of opportunistic pathogenic bacteria. This study provides a direction for the prevention and treatment of lipid metabolic diseases and ideas for the development of marine functional food.

**IMPORTANCE:**

Abnormal lipid metabolism is manifested in a variety of metabolic diseases. Marine algae polysaccharides, such as sodium alginate and its oligosaccharides, can reduce blood lipids and improve lipid metabolism to reduce body weight. Polysaccharides are difficult to absorb by the human body due to their large molecular weight, which limits their application in the field of medicine and food. After feeding mice with sodium alginate, agar, and its oligosaccharides, the level of body weight, serum lipids, inflammatory factors, and the pathological damage of liver and intestine were relieved. Gut microbiota analysis showed that two kinds of polysaccharides and their oligosaccharides could improve the composition of gut microbiota at the phylum and genus level, increase the abundance of *Lactobacillus* and *Ackermania*, and reduce the abundance of harmful bacteria. This study provides a direction for the prevention and treatment of lipid metabolic diseases and ideas for the development of marine functional food.

## INTRODUCTION

**Fig 1:**
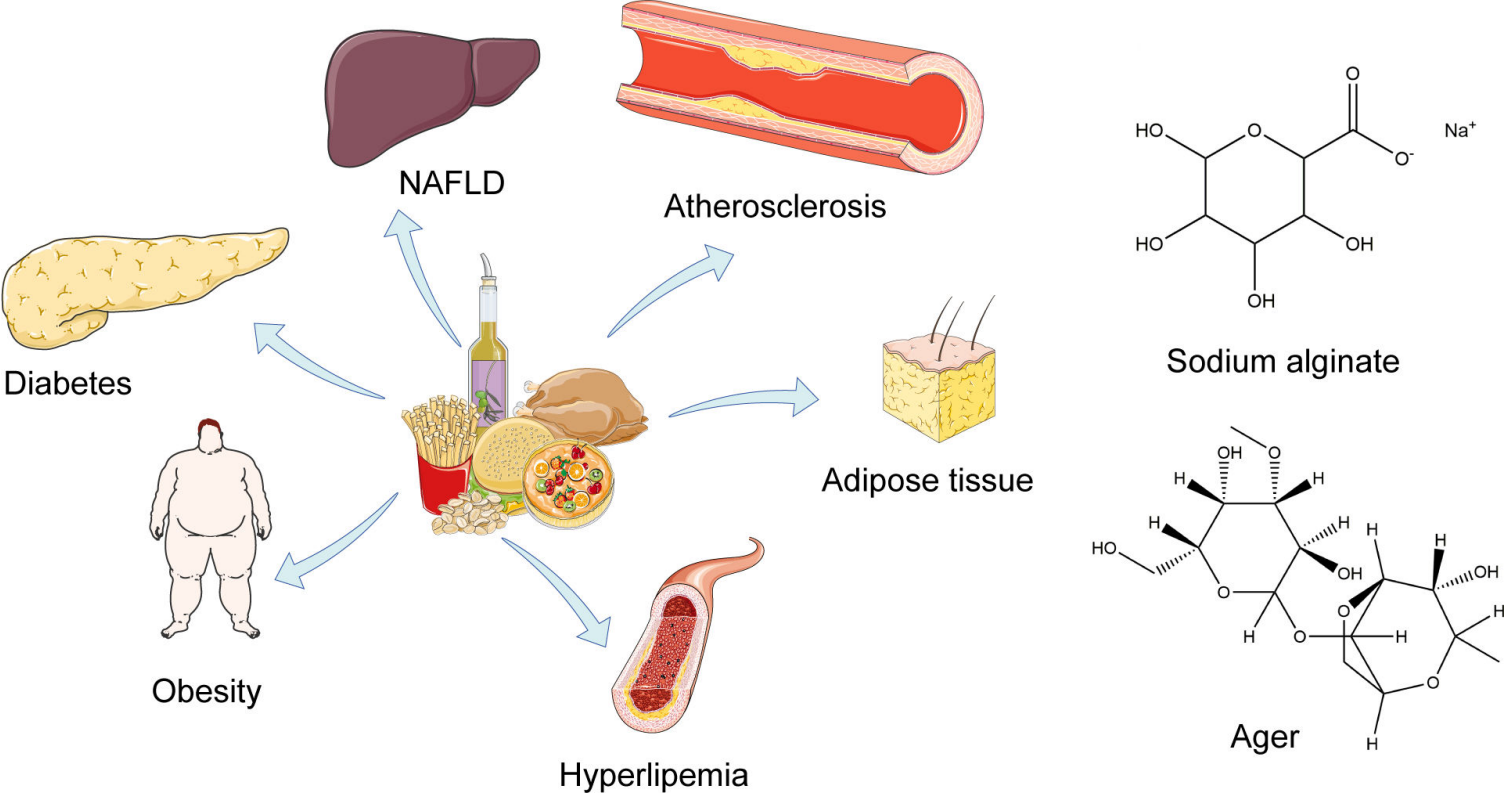
Diseases with lipid metabolism disorder caused by a high-fat diet.

Lipid metabolism is a complex biological process, including nutrient regulation, hormone regulation, and homeostasis regulation. Both unhealthy lifestyle and chronic excess nutrition can cause dysregulation of lipid metabolism, thus leading to more serious lipid-related diseases, including hyperlipidemia, hyperglycemia, obesity, non-alcoholic fatty liver disease (NAFLD), type 2 diabetes mellitus (T2DM), or cancer (1–4) (Fig. 1). Hyperlipidemia is characterized by low levels of high-density lipoprotein (HDL) and high levels of low-density lipoprotein (LDL), triglycerides (TG), and total cholesterol (TC) (5). High concentrations of TC and TG will be deposited on the blood vessel wall and increase the risk of atherosclerosis (6). High levels of LDL and low levels of HDL are closely related to T2DM (7).

Sodium alginate is a naturally occurring anionic polymer that is most commonly isolated from brown algae. It is an acidic polysaccharide that belongs to the linear (unbranched) non-repeating copolymer family. It consists of the variable β-D-mannouronic acid and its C5-terminal, α-L-gulonuronic acid, which are connected by the β-1, 4-glucoside bond. Agar is a kind of polysaccharide extracted from the cell wall of red algae. It is composed of agarose and sulfur agarose. The linear molecule consists of repeated α-D-galactose and 3,6-ether-β-l-galactose alternating links. Some contain low amounts of sulfate. Sulfur agarose contains the same repetitive disaccharide units as agarose, in which some of the hydroxyl groups are replaced by methyl groups or some residues. In this study, agarose oligosaccharides were obtained by enzymolysis from a strain of agar-degrading agar screened from seaweed in our laboratory. Sodium alginate oligosaccharides are obtained by physical, chemical, or biological degradation (8). Studies have shown that sodium alginate oligosaccharides have anti-tumor (9), anti-oxidation (10), anti-inflammatory (11), antibacterial (12), immunomodulatory effects (13), reducing hypertension (14), lowering blood lipids (15), neuroprotection (11), inhibiting obesity (14), and lowering blood sugar (15). The effect of agar on the reduction of hepatosterol elevation caused by a high cholesterol diet. This effect may be through the direct blocking of cholesterol absorption and adsorption of cholic acid to reduce the amount of hepatoenteric circulation. The liver uses cholesterol to synthesize cholic acid, resulting in a decrease in liver cholesterol content.

There is a significant reciprocal relationship between the gut microbiota and its mammalian host, with the microbiota resistant to ingested pathogens, neutralizing carcinogens, and metabolizing otherwise unavailable lipids and polysaccharides into effective bioactive metabolites (16). The intestinal microenvironment is usually in a state of dynamic balance, formed by the gut microbiota and its metabolites (short chain fatty acids [SCFAs], secondary bile acids, etc.) and intestinal mucosal immunity (17), and if the change exceeds the ability of compensatory adjustment, it may lead to a variety of diseases (18). The main groups of gut microbiota are Bacteroides, Firmicutes, and Actinomyces, as well as Proteus, Clostridium, and Bacillus verrucosa, which exist but are less superficial (19). The increase in the abundance of Firmicutes will promote the production of endotoxins and inflammatory factors and destroy the intestinal barrier, while Bacteroides are negatively correlated with both and can protect the intestinal barrier (20, 21).

In recent years, more and more studies have shown that the occurrence of these diseases is closely related to gut microbiota (22–24). Because of its large molecular weight, seaweed polysaccharide is generally considered to be difficult to absorb by the human body, thus limiting its application in the field of medicine and food. Therefore, in this study, polysaccharide is usually degraded into oligosaccharides with relatively small molecular weights. However, whether polysaccharides and oligosaccharides play the same role in improving lipid metabolism and whether the mechanism of action is the same has not been systematically reported. The effects of sodium alginate, agar, and oligosaccharides on body weight, lipid metabolism, secretion of inflammatory factors, intestinal health, and microflora in high-fat diet mice were systematically studied in this study.

## MATERIALS AND METHODS

### Sample preparation

Sodium alginate (Sinopod Group Chemical Reagents Co., LTD, China) was dissolved in 0.3% hydrochloric acid and prepared into a mixture of sodium alginate and hydrochloric acid with a mass fraction of 1.5%. The mixture was stirred until dissolved and degraded at 90°C for 24 h. Centrifuge and retain the supernatant to make the medium and neutralize (pH 7.0). After the supernatant was desalted by dialysis, the sodium alginate oligosaccharide powder was obtained by spray drying.

The bacteria (1 mL) preserved in the laboratory (stored in glycerol) was inoculated into the liquid medium (50 mL/250 mL, protein 10 g/L, yeast extract 5 g/L, sodium chloride 10 g/L), incubated in a shaking table air bath at 150 RPM/min at 30°C and activated for 24 h. After that, the bacterial solution was removed and coated on the solid medium for further culture for 16 h for strain purification. The purified strains were inoculated into 250 mL triangular bottles containing 100 mL fermentation medium and cultured for 20 h. When the fermentation liquid is removed from the bacteria at 4,000 rpm/min at 4°C and 30 min, the supernatant is the crude enzyme liquid. With 0.5% AGAR solution as the substrate of the enzymatic hydrolysis reaction, the diluent of the crude enzyme solution and the substrate are pressed 1:1 mix, degraded at 35°C for 48 h, boiled and terminated after the reaction, rotated evaporation at 60°C for concentration under reduced pressure, centrifuged at 4,000 r/min for 10 min to remove undegraded fragments, appropriate volume of ethanol precipitation to remove enzyme proteins, dialysis to remove small molecular impurities such as salts, freeze drying, and obtain relatively pure agalooligosaccharides, as a follow-up test material.

### Animal model medication and sample collection

C57BL/6 mice (60, half male and half female, 20–25 g, 8–10 weeks of age) were purchased from Liaoning Changsheng Biotechnology Co., LTD, SPF clean grade. Mice were fed at 23°C ± 2°C, humidity at 55% ± 5%, a light and dark cycle for 12 h, and then fed and drank freely for 1 week. One week later, they were randomly divided into 6 groups with 12 animals in each group. Control group (CON) was fed an ordinary diet. High-fat model group (HFD) was fed a high-fat diet. Sodium alginate group (SA) was fed a high-fat diet and gavage sodium alginate (100 mg/kg); sodium alginate oligosaccharide group (SAO) was fed a high-fat diet and intragastrically given sodium alginate oligosaccharide (100 mg/kg); Agar group (Agar) was fed a high-fat diet and gavage agar (100 mg/kg); agar-oligosaccharide group (AOs) was fed a high-fat diet and gavage agar-oligosaccharide (100 mg/kg). After 7 weeks of feeding, the mice were weighed every 2 days. After the mice were anesthetized by intraperitoneal injection, blood was taken from the eyeballs and fixed on the operating table. Feces were collected into a sterile centrifuge tube and stored in a −80°C refrigerator. The heart was perfused with pre-cooled normal saline until the liver turned white, and the liver and intestinal tissues were removed after perfusion with 4% paraformaldehyde for about 30 min and fixed in 4% paraformaldehyde for more than 6 h.

### Determination of serum biochemical indexes

The blood was resting at 4°C, centrifuged at 5,000 rpm at 4°C for 10 min after coagulation, and the supernatant was taken as a serum sample, which was strictly operated according to the TC, TG, HDL-C, LDL-C test kit (Nanjing Jiancheng Institute of Biological Engineering, China). Serum levels of tumor necrosis factor (TNF-α), interleukin-1β (IL-1β), and interleukin-6 (IL-6) were determined by an enzyme-linked immunosorbent assay kit (Nanjing Institute of Jiancheng Bioengineering, China).

### Pathological examination

The mouse tissues were dehydrated and embedded in paraffin wax to make wax blocks, and the tissue sections were prepared. After drying, the slices were baked at 60°C for 2 h. After dewaxing, the sections were stained with hematoxylin, separated by hydrochloric acid and alcohol, returned to blue with 1% ammonia, dyed with eosin and dehydrated, sealed with optical gum, observed, and photographed under a microscope.

### Gut microbiota 16S rRNA sequencing

The extraction of intestinal microbial DNA was performed according to the instructions of the fecal genome DNA extraction kit (DE-05715, FOREGENE, China), and the total DNA of each sample was extracted separately and frozen at −80°C for future use. The V4 variable region was amplified by PCR using the universal primer Forward: 515R-GTGCCAGCMGCCGCGGTAA; Reverse: 806R-GGACTACHVGGGTWTCTAAT (25). The length of the amplified product was 430 bp, and the sequencing junction was connected to construct the sequencing library of the V4 region. The original image data files generated by Illumina Miseq/Hiseq are converted to Sequenced Reads by Base Calling analysis. The original data obtained from the sequencing results were processed, the low-quality sequences were removed, and the high-quality sequences were collected for subsequent analysis. The splice sequence was removed from the original sequencing data, and the double-ended sequence was spliced to form a single sequence. Low-quality sequences and unmatched sequences were filtered out and compared against the 16s rDNA database. The paired-end (PE) reads obtained by sequencing were first spliced according to overlap, then the sequence quality was controlled and filtered after the samples were distinguished, and then operational taxonomic unit (OTU) clustering (amplicon sequence variant [ASV] denoising) analysis and species taxonomic analysis were carried out. Based on OTU clustering (ASV denoising) analysis results, the OTU (ASV) diversity index can be analyzed, and sequencing depth can be detected. Based on taxonomic information, statistical analysis of community structure can be performed at various taxonomic levels. On the basis of the above analysis, a series of in-depth statistical and visual analyses can be performed on the community composition and phylogenetic information of diverse species, such as Beta diversity analysis, grouping test analysis, difference significance test, environmental factor association analysis, association and model prediction analysis, and functional prediction.

### Statistical analysis

SPSS 23.0 software was used for statistical analysis, and the calculated values were expressed as Mean ± SEM. One-way ANOVA test was used to conduct inter-group statistical analysis of the data, using *P* < 0.05 was considered statistically significant.

## RESULTS

### Effects of two kinds of algal polysaccharides and their oligosaccharides on the body weight of hyperlipidemia mice

**Fig 2:**
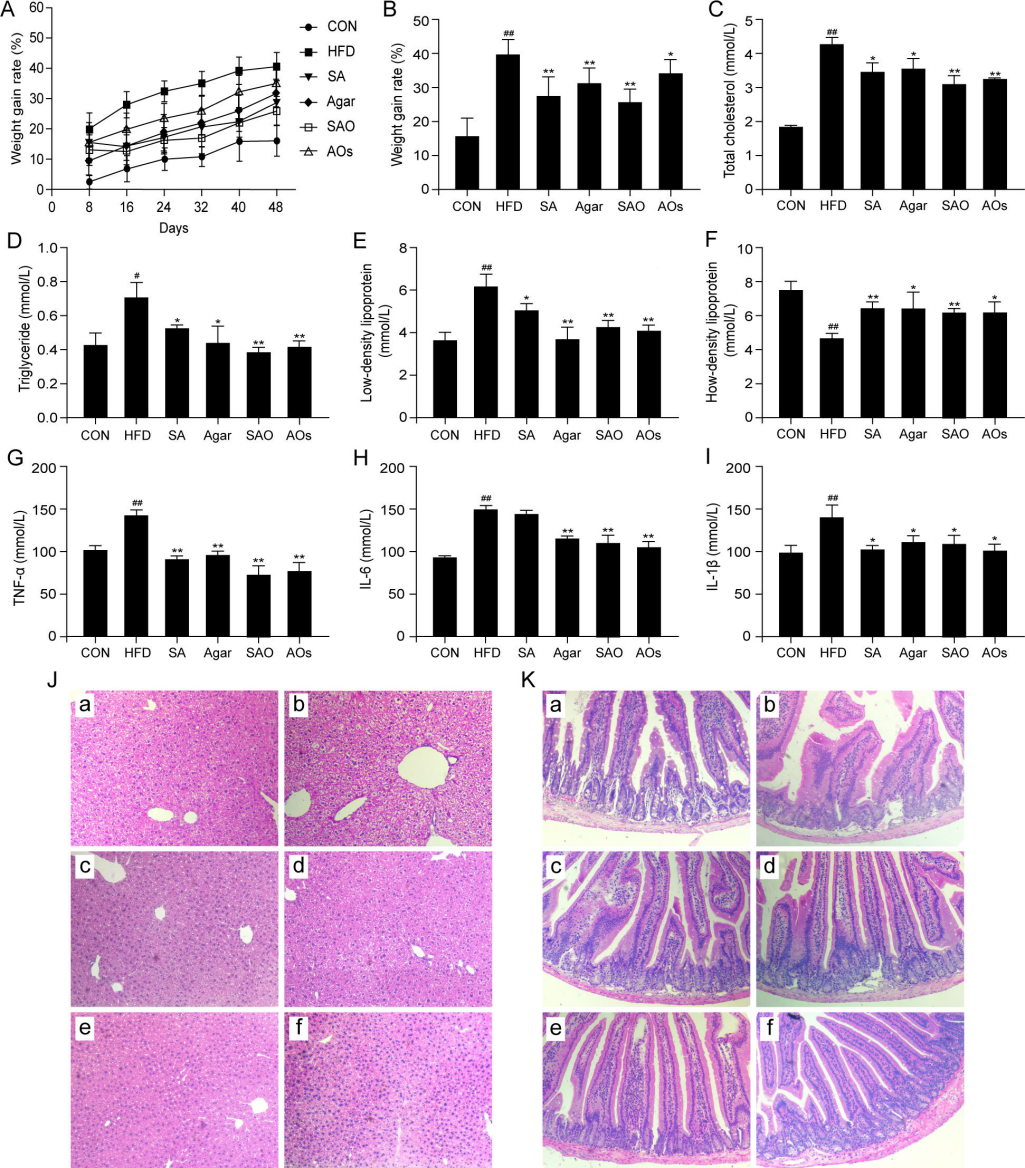
Effects of three kinds of seaweed polysaccharides and their oligosaccharides on lipid metabolism in hyperlipidemia mice. (A) Changes in body weight of mice (*n* = 6); (B) weight growth rate of mice after 7 weeks of feeding; (C) the expression of total cholesterol in blood; (D) the expression of triglyceride in blood; (E) the expression of low-density lipoprotein in blood; (F) the expression of high-density lipoprotein in blood; (G) the expression of TNF-α in the blood; (H) the expression of IL-6 in the blood; (I) the expression of IL-1β in the blood; (J) representative images of the liver tissue structure detected by hematoxylin and eosin (H&E) staining (100×); (K) representative images of the intestinal tract tissue structure detected by H&E staining (100×). ##, *P <* 0.01 vs CON; *, *P* < 0.05; **, *P <* 0.01 vs HFD. CON group was fed an ordinary diet. HFD group was fed a high-fat diet. SA group was fed a high-fat diet and gavage sodium alginate (100 mg/kg); SAO group was fed a high-fat diet and intragastrically given sodium alginate oligosaccharide (100 mg/kg); Agar group (Agar) was fed a high-fat diet and gavage agar (100 mg/kg); AOs group was fed a high-fat diet and gavage agar-oligosaccharide (100 mg/kg). All the groups were treated every other day.

In the experiment, the weight of the mice was measured every 2 days. As shown in Fig. 2A, the weight of the HFD group increased the fastest, and the growth rate (vs first day) was always higher than other groups. The weight growth rate of mice given two kinds of algal polysaccharides and oligosaccharides by intragastric administration was lower than that of the HFD group and slightly higher than that of the CON group. After 7 weeks of experiment, the weight of mice was shown in Fig. 2B. The weight of mice in the CON group was the lightest, and the weight of mice in the HFD group was the heaviest. The weight of mice in the two seaweed polysaccharide and oligosaccharide groups was between the two.

### Effects of two kinds of seaweed polysaccharides and their oligosaccharides on blood lipids in mice with a high-fat diet

High-fat diet can lead to an increase in TG and TC content in blood, thus leading to a series of metabolic diseases (26). Too much LDL-C is considered harmful to health and can inhibit the production of atherosclerosis (27). The effects of two kinds of seaweed polysaccharides and their oligosaccharides on the blood total TC level of mice on a high-fat diet were shown in Fig. 2C. Compared with the CON group, the blood total cholesterol level of mice in the HFD group was increased by 2.42 mmol/L. The results of blood TG levels of mice on a high-fat diet were shown in Fig. 2D. Compared with the CON group, the blood triglyceride level of mice in the HFD group was increased by 0.28 mmol/L. Both kinds of alginate polysaccharides and oligosaccharides can significantly reduce the content of triglycerides in the blood of high-fat diet mice, and the effect of oligosaccharides is better than that of polysaccharides, and the effect of sodium alginate and oligosaccharides is better than that of agar and oligosaccharides. The results of LDL-C in the blood of mice on a high-fat diet were shown in Fig. 2E. Compared with the CON group, the LDL-C level in the blood of mice in the HFD group was significantly increased, while the two kinds of seaweed polysaccharides and their oligosaccharides could significantly reduce the level of LDL-C in the blood of mice on a high-fat diet. The effect of sodium alginate was better than that of its oligosaccharides, and the effect of AGAR was worse than that of its oligosaccharides. The results of the influence of HDL-C levels in the blood of mice on a high-fat diet are shown in Fig. 2F. Compared with the CON group, the level of HDL-C in the blood of mice in the HFD group is significantly decreased (by 2.82 mmol/L), while both kinds of algal polysaccharides and their oligosaccharides can increase the level of HDL-C in the blood of mice on a high-fat diet.

### Effects of two kinds of algal polysaccharides and their oligosaccharides on the levels of blood inflammatory factors in high-fat diet mice

The expression results of TNF-α in the blood of high-fat diet mice were shown in Fig. 2G. Compared with the CON group, the level of TNF-α in the blood of HFD group mice was significantly increased (40.96 mmol/L), while both kinds of algal polysaccharides and their oligosaccharides could reduce the level of TNF-α in the blood of high-fat diet mice. The effect of oligosaccharides is better than that of polysaccharides. The expression results of IL-6 in the blood of high-fat diet mice were shown in Fig. 2H. Compared with the CON group, the level of IL-6 in the blood of HFD group mice was extremely significantly increased (53.19 mmol/L), while both kinds of algal polysaccharides and their oligosaccharides could reduce the level of IL-6 in the blood of high-fat diet mice. The expression of IL-1β in the blood of mice on a high-fat diet was shown in Fig. 2I. Compared with the CON group, the level of IL-1β in the blood of mice in the HFD group was significantly increased (41.28 mmol/L), and both kinds of algal polysaccharides and their oligosaccharides could significantly reduce the level of IL-1β in the blood of mice on a high-fat diet.

### Effects of two kinds of algal polysaccharides and their oligosaccharides on liver and small intestine morphology in high-fat diet mice

The liver is one of the most important places for lipid metabolism in the body. Since the synthesis and metabolism of lipids in the liver are in a state of dynamic balance, once the lipid metabolism is abnormal, it will lead to the accumulation of lipids in the liver, which will be stored in liver cells and form lipid droplets of different sizes, thus forming fatty liver, which may cause other metabolic diseases in severe cases (28). As shown in Fig. 2J, only a small number of small lipid droplets existed in the liver cells of mice in the CON group. In the liver cells of HFD mice, the number and volume of lipid droplets increased significantly, and lipid droplets occupied almost the entire cytoplasm of the liver cells. After the intervention of two kinds of seaweed polysaccharides and their oligosaccharides, the volume and quantity of fat droplets in the liver of model mice were reduced, which could significantly inhibit the formation of fat droplets in the liver.

There are a large number of intestinal villi with a complex anatomical structure in the intestine of the body. The intestinal villi are composed of a single layer of epithelial cells and a large number of capillaries, which are an important organ for the body to absorb nutrients and drugs. Once the intestinal villi are destroyed, it will have a serious impact on the absorption and metabolism of some substances in the body, which will lead to metabolic diseases and even induce cancer. As shown in Fig. 2K, the intestinal villi of mice in the CON group were neatly arranged, and the intestinal villous epithelial cells were all monolayer cells. The intestinal villi morphology of the HFD group mice was seriously damaged, and there was an accumulation of intestinal villi epithelial cells. After the intervention of two kinds of algae polysaccharides and their oligosaccharides, it can be seen that the intestinal villi structure of the Agar and SAO group mice was relatively clear, and the epithelial cells were arranged neatly, but a large number of cells existed in the chylous tube. In the SA and AOs groups, the intestinal tract of mice could recover to the level of normal mice, the intestinal villi were arranged neatly, and the intestinal villi epithelial cells were monolayer cells.

### Quality evaluation of gut microbiota sequencing results and OTU cluster analysis

**Fig 3:**
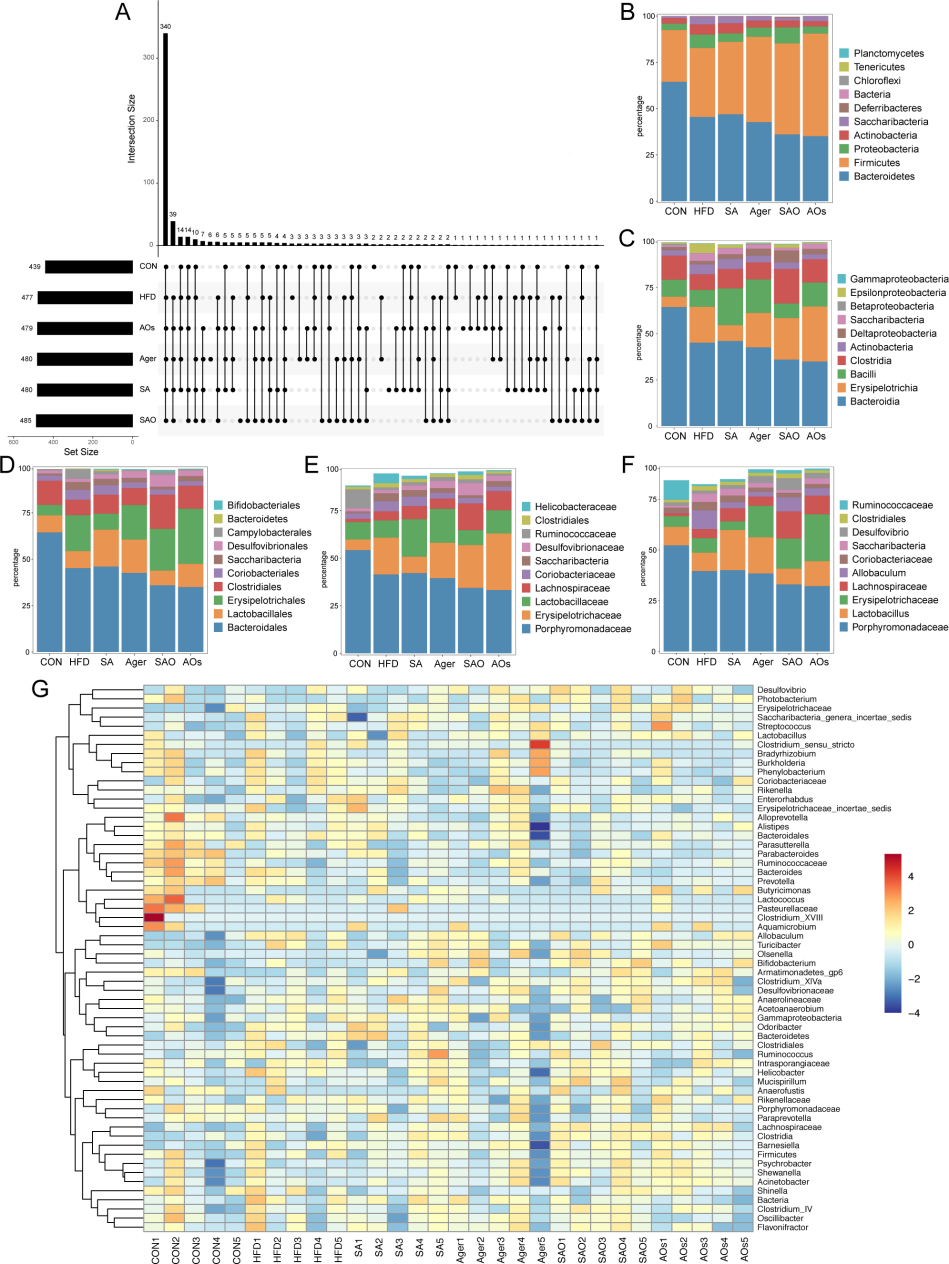
Cluster analysis of OTU (*n* = 6). (A) UpSet plot of the classification results of OTU. Each circle represented a group; (B) cluster analysis of OTU at the Phylum level. Plot the top 10 species according to their abundance scale; (C) cluster analysis of OTU at the Class level. Plot the top 10 species according to their abundance scale; (D) cluster analysis of OTU at the Order level. Plot the top 10 species according to their abundance scale; (E) cluster analysis of OTU at the Family level. Plot the top 10 species according to their abundance scale; (F) cluster analysis of OTU at the Genus level. Plot the top 10 species according to their abundance scale; (G) classification and abundance of gut microbiota at the Genus level. Species in the samples were ranked according to abundance, and the top 60 genera were clustered. *, *P* < 0.05 vs CON. CON group was fed an ordinary diet. HFD group was fed a high-fat diet. SA group was fed a high-fat diet and gavage sodium alginate (100 mg/kg); SAO group was fed a high-fat diet and intragastrically given sodium alginate oligosaccharide (100 mg/kg); Agar group (Agar) was fed a high-fat diet and gavage agar (100 mg/kg); AOs group was fed a high-fat diet and gavage agar-oligosaccharide (100 mg/kg). All the groups were treated every other day.

This experiment was divided into six groups, each of which randomly selected five fecal samples to extract genomic DNA and sequenced. Through the steps of library construction and computer sequencing, a total of 1,456,191 high-quality sequences were statistically generated, with an average of 48,539.7 sequences generated per sample. QIIME software (version 1.8.0) was used to process the original sequencing data (29, 30), and according to different similarity levels, all sequences were classified into OTUs. Usually, biological information statistics were performed on OTUs at a 97% similarity level. The sequence information obtained through OTU cluster analysis was compared with the database Silva to obtain the classification information of specific microbial species, and then the composition and proportion of microorganisms were analyzed and counted, and the composition of microbial communities of sample species was counted at the level of phyla, class, order, family, genus, and species at the taxonomic level. After OTU cluster analysis, an UpSet plot was drawn for the OTU classification results of each group, as shown in Fig. 3A. The total number of OTUs in the 6 groups is 340. There were 39 different OTU sequences between the experimental group and the CON group. There were seven different OTU sequences in the two groups treated with algal polysaccharides and oligosaccharides.

As shown in Fig. 3B, in the classification level analysis of Phylum, the bar chart was drawn according to the abundance ratio of species. The top three species in the CON group were *Bacteroidetes* (64.40%), *Firmicutes* (28.02%), and *Proteobacteria* (3.45%). *Bacteroidetes* (45.43%), *Firmicutes* (37.24%), and *Proteobacteria* (7.31%) in the HFD group were *Bacteroidetes* (45.43%), and the comparison between the two groups showed that the proportion of *Bacteroidetes* in the preparation process of the HFD animal model was obviously reduced. But the proportion of *Bacteroidetes* and *Firmicutes* increased. The top three species in the SA group were *Bacteroidetes* (46.87%), *Firmicutes* (39.19%), and *Actinobacteria* (5.50%). The Agar group was *Firmicutes* (46.05%), *Bacteroidetes* (42.66%), and *Proteobacteria* (5.07%). The SAO group was *Firmicutes* (49.20%), *Bacteroidetes* (36.02%), and *Proteobacteria* (8.66%). The AOs group was *Firmicutes* (55.40%), *Bacteroidetes* (35.05%), and *Proteobacteria* (3.88%). Compared with other groups, *Bacteroidetes* in the CON group were more than 50% and can be regarded as the dominant *Bacteroidetes*, while *Bacteroidetes* in the HFD group were decreasing. After being treated with two kinds of polysaccharides and oligosaccharides, *Bacteroidetes* in mouse faeces could be further reduced. The proportion of *Firmicutes* and *Proteobacteria* in the drug action group was increased. In group SA, the abundance of *Actinobacteria* exceeds that of *Proteobacteria*.

As shown in Fig. 3C, at the Class classification level, a bar chart was drawn according to the abundance ratio of species. *Bacteroidia* (64.36%), *Clostridia* (13.00%), and *Bacilli* (9.23%) were the top three species in the CON group. *Bacteroidia* (45.11%), *Erysipelotrichia* (19.40%), and *Bacilli* (9.17%) in the HFD group were *Bacteroidia* (45.11%) and *Bacilli* (9.17%). The top three species in the SA group were *Bacteroidia* (45.97%), *Bacilli* (19.93%), and *Clostridia* (10.50%). The Agar group was *Bacteroidia* (42.55%), *Erysipelotrichia* (18.65%), and *Bacilli* (18.16%). The SAO group was *Bacteroidia* (35.91%), *Erysipelotrichia* (22.53%), and *Clostridia* (18.64%). *Bacteroidia* (34.91%), *Erysipelotrichia* (29.76%), and *Bacilli* (12.98%) in the AOs group were *Bacteroidia*, *Erysipelotrichia,* and *Bacilli*. In the CON group, the proportion of *Bacteroidia* was 64.36%, while in the HFD group, the proportion decreased to 45.11%. In the animal models treated with polysaccharides and oligosaccharides, the abundance of *Bacteroidia* decreased, and the proportion of *Erysipelotrichia* increased. The proportion of *Erysipelotrichia* in the SA group was similar to that in the CON group.

As shown in Fig. 3D, at the Order classification level, the analysis draws a histogram based on the abundance ratio of species. The top three species in the CON group are *Bacteroidales* (64.36%), *Clostridiales* (12.93%), and *Lactobacillales* (9.22%). In the HFD group, *Bacteroidales* (45.11%), *Erysipelotrichales* (19.40%), and *Lactobacillales* (9.16%) were present. The top three species in the SA group are *Bacteroidales* (45.97%), *Lactobacillales* (19.91%), and *Clostridiales* (10.22%). The Agar group is *Bacteroidales* (42.55%), *Erysipelotrichales* (18.65%), and *Lactobacillales* (17.95%). The SAO group was *Bacteroidales* (35.91%), *Erysipelotrichales* (22.53%), and *Clostridiales* (18.32%). The Agar group was *Bacteroidales* (34.91%), *Erysipelotrichales* (29.76%), and *Lactobacillales* (12.51%). The dominant species in the CON group was *Bacteroidales*, the abundance ratio of which was higher than that of the HFD group and the drug-treated group. The *Erysipelotrichales* abundance in the HFD group was higher than that in the CON group, and the *Erysipelotrichales* abundance in the SA group was similar to that in the CON group, while the abundance of *Lactobacillales* was increased.

As shown in Fig. 3E, at the level of Family classification, the analysis plots a bar chart based on the abundance ratio of species. Among them, the top three species in the CON group were *Porphyromonadaceae* (54.16%), *Ruminococcaceae* (9.92%), and *Lactobacillaceae* (9.16%). HFD group consisted of *Porphyromonadaceae* (41.38%), *Erysipelotrichaceae* (19.40%), and *Lactobacillaceae* (9.07%). The top three species in the SA group were *Porphyromonadaceae* (42.11%), *Lactobacillaceae* (19.77%), and *Erysipelotrichaceae* (8.59%). The Agar group consists of *Porphyromonadaceae* (39.44%), *Erysipelotrichaceae* (18.65%), and *Lactobacillaceae* (17.83%). The SAO group was composed of *Porphyromonadaceae* (34.34%), *Erysipelotrichaceae* (22.53%), and *Lachnospiraceae* (14.03%). The AOs group was composed of *Porphyromonadaceae* (33.19%), *Erysipelotrichaceae* (29.76%), and *Lactobacillaceae* (12.23%). The highest abundance species in the CON group was *Porphyromonadaceae*, and the abundance of *Porphyromonadaceae* in other groups decreased to varying degrees, among which the proportion of *Porphyromonadaceae* in the AOs group was the lowest, and it was 33.19%. The abundance and proportion of *Erysipelotrichaceae* and *Lactobacillaceae* in each group affected by the drug have increased. In addition, *Lachnospiraceae* and *Coriobacteriaceae* have increased to varying degrees. The abundance of *Ruminococcaceae* decreased compared with the CON group.

As shown in Fig. 3F, at the Genus classification level, the analysis plots a bar chart based on the abundance ratio of species. Among them, the top three species in the CON group are unclassified_*Porphyromonadaceae* (52.23%), *Ruminococcaceae* (9.62%), and *Lactobacillaceae* (9.16%). HFD group consisted of *Porphyromonadaceae* (39.58%), *Allobaculum* (9.23%), and *Lactobacillaceae* (9.07%). The top three species in the SA group were *Porphyromonadaceae* (40.04%), *Lactobacillaceae* (19.77%), and *Lachnospiraceae* (6.37%). The Agar group consists of *Porphyromonadaceae* (38.40%), *Lactobacillaceae* (17.83%), and *Erysipelotrichaceae* (15.51%). The SAO group was composed of *Porphyromonadaceae* (32.98%), *Erysipelotrichaceae* (14.83%), and *Lachnospiraceae* (13.31%). The AOs group was composed of *Porphyromonadaceae* (32.17%), *Erysipelotrichaceae* (23.15%), and *Lactobacillaceae* (12.23%). The highest abundance species in the CON group was *Porphyromonadaceae*, and the abundance of *Porphyromonadaceae* in other groups decreased to varying degrees.

In order to further determine the direct evolutionary correlation between the effects of the two kinds of seaweed polysaccharides and oligosaccharides on the gut microbiota, and to determine the possible modes of drug action. At the level of genus classification, species in the sample were sorted according to their abundance, and cluster analysis was performed on the top 60 genera with the abundance ranking. The results were shown in Fig. 3G. In the figure, each microbial species in the sample was represented by a corresponding color block. The higher the abundance, the closer the color of the block was to red; the lower the abundance, the closer the color was to dark blue. In this way, the similarity between samples, the clustering relationship between species, and the similarity of community composition were analyzed in detail, where the length of the branches represented the distance between samples.

### Sample Alpha and Beta diversity analysis

**Fig 4:**
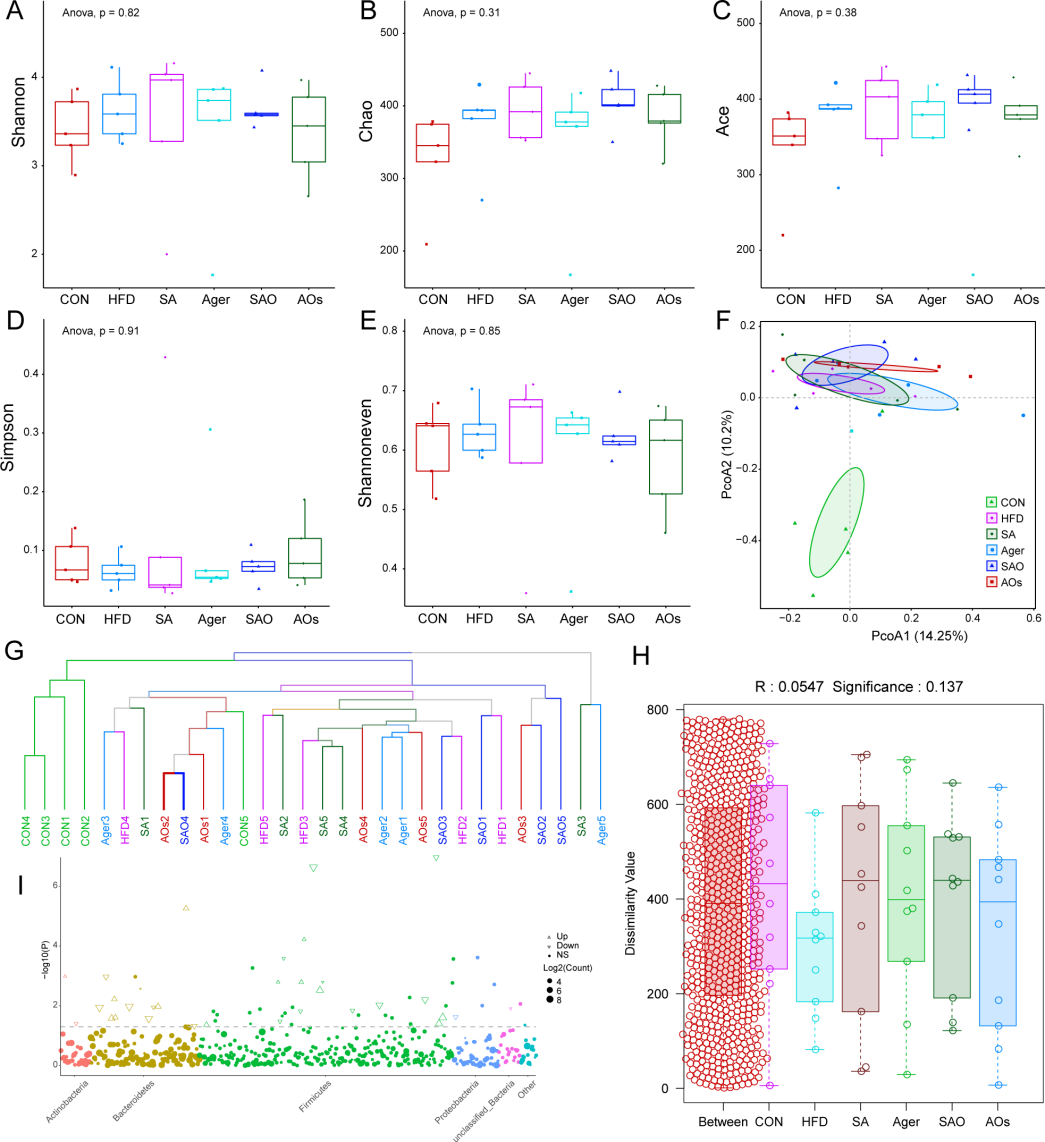
Alpha diversity curve and Beta diversity analysis. (A) Alpha diversity box-plot of Shannon; (B) Alpha diversity box-plot of Chao; (C) Alpha diversity box-plot of Ace; (D) Alpha diversity box-plot of Simpson; (E) Alpha diversity box-plot of Shannoneven; (F) principal coordinates analysis (PCoA) results of each sample. PcoA1 and PcoA2 are two principal coordinates. PcoA1 represents the principal coordinate that explains the maximum change in the data. PcoA2 is the principal coordinate component that accounts for the largest proportion of the remaining coordinates; (G) clustering relationship between samples. The similarity of samples and their clustering relationship with species and community structure similarity were analyzed; (H) similarity analysis. The Between box represents the distance value of the difference between the groups, and the other boxes represent the difference within the respective groups. (I) MetagenomeSeq analysis of differences between groups of samples. The horizontal axis is displayed in horizontal alphabetical order of taxonomic phyla, with different colors representing different phyla. CON group was fed an ordinary diet. HFD group was fed a high-fat diet. SA group was fed a high-fat diet and gavage sodium alginate (100 mg/kg); SAO group was fed a high-fat diet and intragastrically given sodium alginate oligosaccharide (100 mg/kg); Agar group (Agar) was fed a high-fat diet and gavage agar (100 mg/kg); AOs group was fed a high-fat diet and gavage agar-oligosaccharide (100 mg/kg). All the groups were treated every other day.

Microbial diversity is studied in community ecology, and the abundance and diversity of microbial communities can be reflected through sample diversity analysis (Alpha diversity), including a series of statistical analysis indices to estimate the species abundance and diversity of environmental communities. The commonly used analysis indicators of Alpha diversity include Shannon, Chao, Ace, Simpson, Shannoneven, and Goods coverage, etc. (Fig. 4A through E). The Shannon index and Simpson index represent the community diversity in the sample, and their values are affected by the abundance and evenness of species in the sample. When the abundance of species is certain, the evenness of species in the sample is proportional to the diversity of species. That is, the larger the Shannon index and Simpson index are, the higher the species evenness and diversity of the sample. Chao index, Ace index, and Goods coverage index represent the total number of microbial species in the tested sample. The higher the value, the greater the number of species, and whether the sequencing depth has covered all species in the sample. The Goods coverage index in each group was close to 1, indicating that the sequencing results were of high quality and could cover almost all species in the samples. The species diversity of the HFD group was higher than that of the CON group. After two kinds of seaweed polysaccharides and oligosaccharides were added to the diet, the species diversity of gut microbiota of mice was basically the same as that of the HFD group, but had certain differences from the CON group. The results showed that dietary supplementation of two kinds of seaweed polysaccharides and their oligosaccharides had no statistical difference on the species richness and diversity of intestinal microflora in mice (*P* < 0.05).

Beta diversity analysis is a method to study the similarity of the overall community composition in samples, which is used to analyze the differences in species diversity in different samples. Principal coordinates analysis (PCoA) is used to calculate the individual differences among samples and analyze the sample duplication within a group. The Weighted UniFrac algorithm was used to compare the species composition of microorganisms in each group. The method also calculated the species and abundance changes of the species. The sample distance represented the difference in the species and quantity of OTU between the communities, as shown in Fig. 4F. Its species diversity and abundance are high. The other groups were closer together, suggesting that their microbial communities were more similar in composition. Hierarchical clustering analysis was carried out on the distance matrix, a tree structure was constructed, and the tree relationship form was obtained for visual analysis (Fig. 4G). The length of the branches represents the distance between the samples; the more similar the samples, the closer together they will be. According to the clustering diagram, the samples in the CON group are clustered in a cluster, which has a high degree of similarity. The samples of mice fed a high-fat diet were relatively clustered, suggesting that a high-fat diet had a certain effect on gut microbiota.

### Sample species similarity analysis

Similarity analysis is a non-parametric test used to determine whether the differences between groups are significantly greater than the differences within groups, and thus whether the grouping is meaningful. First, all distances are sorted from small to large according to the distance between the two samples, and R values are calculated. As shown in Fig. 4H, the box corresponding to Between represents the distance value of the difference between groups, while the other boxes, respectively, represent the difference within each group. The test results are used to analyze the large difference between groups and the difference within groups. The R value of the CON group and high-fat diet (HFD) group was 0.428 (*P* = 0.02), indicating that a high-fat diet had an effect on the gut microbiota of mice. Compared with the DFH group, the SAO group had the closest R value to 1. The largest difference between the groups was found in SA and AOs. To further analyze the differences between the CON group and HFD group, metagenomeSeq was used to compare the differences between the samples. The Manhattan display of metagenomeSeq significant difference analysis results is shown in Fig. 4I. The horizontal axis is displayed in horizontal alphabetical order of taxonomic phyla, with different colors representing different phyla. Each dot represents one OTU, and the size represents the average abundance value. According to Log2FC absolute value greater than 1 and *P* value less than 0.05, OTUs were divided into three categories: up-regulated, down-regulated, and non-significant, and marked with different shapes.

### Classification analysis of community phenotype

**Fig 5:**
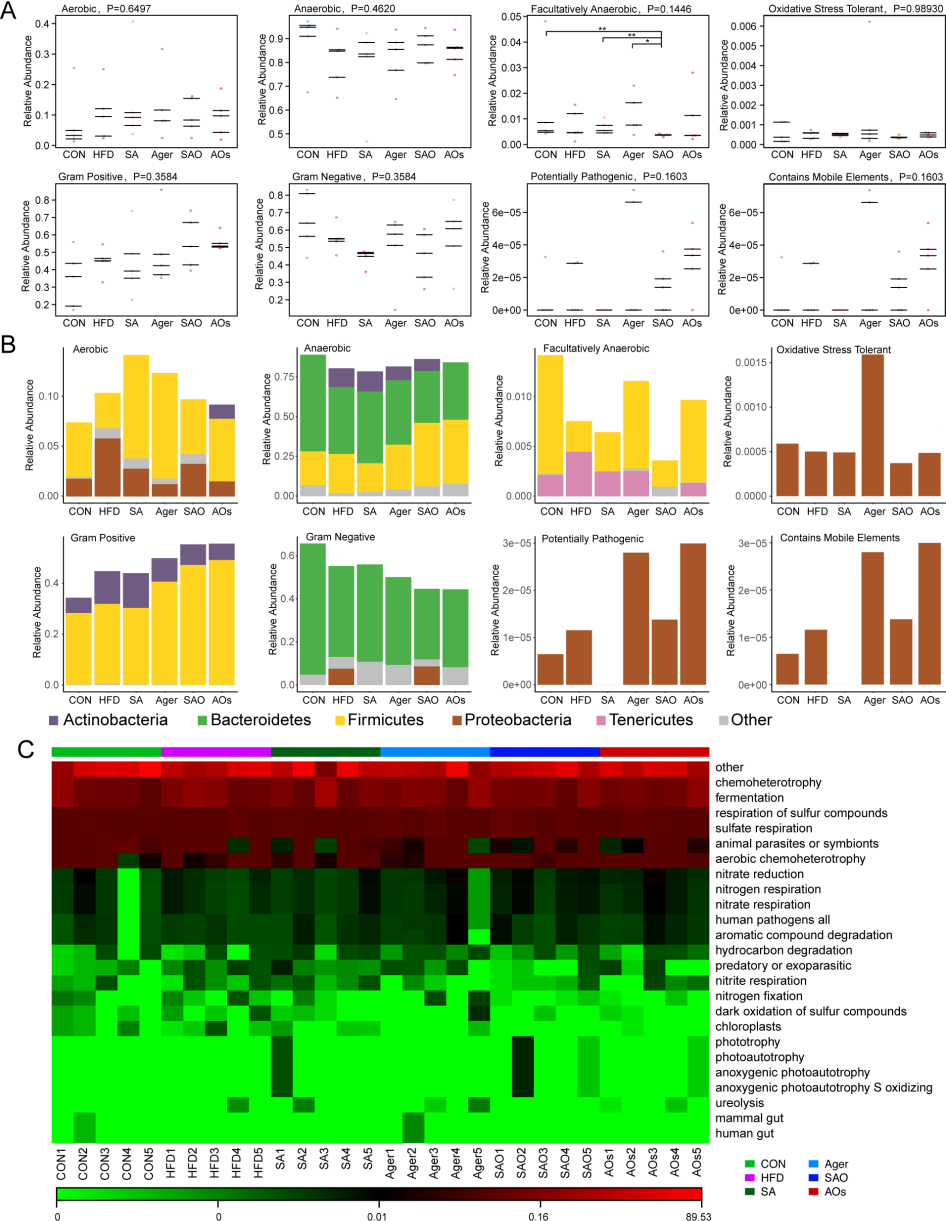
Classification analysis of community phenotype. (A) BugBase was used to predict the proportion of aerobic, anaerobic, facultative anaerobic, potential pathogenicity, oxidative stress tolerance, and contains mobile elements; (B) OTU contribution map of relative abundance of phyla in each phenotype; (C) functional abundance heat map. *, *P <* 0.05; **, *P <* 0.01 vs CON. CON group was fed an ordinary diet. HFD group was fed a high-fat diet. SA group was fed a high-fat diet and gavage sodium alginate (100 mg/kg); SAO group was fed a high-fat diet and intragastrically given sodium alginate oligosaccharide (100 mg/kg); Agar group (Agar) was fed a high-fat diet and gavage agar (100 mg/kg); AOs group was fed a high-fat diet and gavage agar-oligosaccharide (100 mg/kg). All the groups were treated every other day.

Using BugBase software (31), microbial communities can be classified according to seven types of phenotypes, including oxygen utilizing, aerobic, anaerobic, facultatively anaerobic, oxidative stress tolerant, Gram-positive, Gram-negative, biofilm forming, pathogenic (pathogenic), mobile element containing, etc. This information can help to better understand the relationship between microbes and disease. As shown in Fig. 5A, after statistics, there was no statistical difference between groups. However, the analysis of facultatively anaerobic results shows that the SAO group is different from the CON group, SA group, and Agar group, respectively. As shown in Fig. 5B, in aerobic water, the main OTU contributing bacteria are *Proteobacteria*, *Firmicutes,* and *Actinobacteria*. Compared with the CON group, the proportion of *Proteobacteria* in the HFD group increased, while the proportion of *Firmicutes* decreased. As for anaerobic bacteria, the main bacterial groups contributing to OTU are *Firmicutes*, *Bacteroidetes,* and *Actinobacteria. Proteobacteria* are the main OTU contributing bacteria in potential pathogenicity prediction. Oxidative stress tolerance (*P* = 0.8930); *Proteobacteria* was the main OTU contributing bacteria in the prediction. In the mobile element containing (*P* = 0.1603), the main OTU contributing bacteria were *Firmicutes*, *Proteobacteria,* and *Verrucobacteria*. Functional Annotation of Prokaryotic Taxa (FAPROTAX) is a functional annotation database for the analysis of microbial community functions based on prokaryotic microbial classification. Contains more than 7,600 functional annotation information collected from more than 4,600 prokaryotic microorganisms in more than 80 functional groups (such as nitrate respiration, methanogenesis, fermentation, plant pathogens, etc.). As shown in Fig. 5A, the functions predicted by FAPROTAX mainly focus on the functions of microorganisms in oceans and lakes, especially the cycling functions of sulfur, carbon, hydrogen, and nitrogen. The functional abundance heat map is shown in Fig. 5C, drawn by the functional abundance matrix. In the figure, each column represents a sample, rows represent functions, and color blocks represent functional abundance values. The more red the color, the higher the abundance, and the more green the color, and vice versa.

## DISCUSSION

Abnormal lipid metabolism can occur in many metabolic diseases, such as hyperlipidemia, hyperglycemia, obesity, NAFLD, T2DM, and cancer (1, 2). In recent years, more and more studies have shown that the occurrence of these diseases is closely related to gut microbiota (22–24). The resident microbiome produces a variety of metabolites that regulate cell function (32). Gut microbiota plays an important role in the pathophysiology of obesity, T2DM, and other diseases of glucose and lipid metabolism (33, 34). Intestinal bacterial diversity and gene richness were reduced in patients with abnormal glucose and lipid metabolism. Gut microbiota transplantation from obese patients can lead to obesity in sterile mice, which proves that obesity with gut microbiota disorder is related to metabolic diseases (35). In addition, inoculation of enterobacteria isolated from the feces of severely obese patients into infertile mice resulted in severe obesity and insulin resistance (36). The analysis also showed that people with low microbial abundance generally had more pro-inflammatory bacteria, most of whom had insulin resistance, high triglyceride levels, and a higher risk of T2DM (37). So far, studies have shown that the structural imbalance of gut microbiota mainly affects the metabolism of sugars and lipids in the body, including SCFAs, bile acids, choline, amino acids, and other metabolites (32). The results of this study showed that after feeding a high-fat diet for 7 weeks, the weight of mice increased significantly, the blood lipid level increased significantly, the formation of fat droplets in liver cells was obvious, the small intestinal microvilli of mice were destroyed, the accumulation of epithelial cells was obvious, and the abundance of beneficial bacteria in the gut microbiota of mice was significantly reduced, while the abundance of harmful bacteria was significantly increased. After feeding the two kinds of algal polysaccharide and its oligosaccharides, the weight gain trend of mice was significantly slowed down, the blood lipid level and the production of inflammatory factors were significantly reduced, and the accumulation of fat in the liver was inhibited, and the intestinal villi morphology was protected, so that it could play a normal function, among which the protective effect of agarose oligosaccharides was the most obvious.

The gut microbiota genera include *Porphyromonadaceae*, *Lactobacillus*, *Helicobacter*, *Alistipes*, and other bacteria. *Allobaculum* and *Akkermansia*, among others. Lactobacillus can reduce body weight, reduce lipid levels and inflammatory cytokines, and inhibit the proliferation of pathogenic bacteria, thus enhancing the host immunity (20). *Akkermansia* is one of the inhibitory bacteria produced by lipopolysaccharide (LPS), which can reduce endotoxemia, protect the intestinal barrier, and improve obesity and diabetes (38). Acetic acid, butyric acid, and propionic acid, metabolites of *Alistipes*, play an important role in inhibiting inflammation. The results of gut microbiota analysis showed that the two kinds of algal polysaccharides and their oligosaccharides could improve the gut microbiota composition of mice at the phylum and genus levels, increase the abundance of probiotics such as *Lactobacillus* and *Ackermannia*, and reduce the abundance of harmful bacteria. Sodium alginate oligosaccharides and agarose oligosaccharides can improve lipid metabolism and slow down weight gain in high-fat diet mice by increasing the abundance of gut microbiota closely related to lipid metabolism. At the level of gut microbiota, *Bacteroidetes* occupy the main advantage and play a protective role in the intestinal tract of mice to prevent the intestinal barrier from being destroyed (21). After the mice were fed a high-fat diet, the level of *Bacteroidetes* in the gut decreased significantly, making the gut more vulnerable to damage. However, after the addition of three kinds of seaweed polysaccharides and oligosaccharides in the diet, *Bacteroidetes* abundance in the intestinal tract of mice was significantly restored, and Agar and SAO could significantly restore the abundance of *Bacteroidetes*. As the second dominant category in the intestinal tract of mice, *Firmicutes* has the opposite effect from *Bacteroidetes*, which can damage the intestinal barrier. After the mice were fed a high-fat diet, the abundance of *Firmicutes* in the intestine increased significantly, causing certain damage to the intestine. The abundance of *Firmicutes* in the intestinal tract of mice was significantly decreased after dietary addition of two kinds of seaweed polysaccharides and their oligosaccharides, among which Agar had the most significant effect on the decrease of *Firmicutes* abundance. *Candidatus*_*Saccharibacteria* and *Proteobacteria* also predominate in the intestine of mice, and the increased abundance of *Candidatus*_*Saccharibacteria* and *Proteobacteria* may cause diarrhea symptoms (17, 39). After feeding a high-fat diet, the abundance of *Candidatus*_*Saccharibacteria* and *Proteobacteria* in the intestine increased significantly, and the intestine was obviously in an unhealthy state. The abundance of *Candidatus*_*Saccharibacteria* and *Proteobacteria* in the gut of mice was significantly reduced after the addition of two kinds of algal polysaccharides and oligosaccharides, even to the level of normal control mice.

At the mouse gut microbiome level, *Lactobacillus*, *Allobaculum,* and *Akkermansia* are three genera closely related to lipid metabolism, with Lactobacillus reducing body weight, reducing lipid levels and inflammatory cytokines, and inhibiting the proliferation of disease-causing bacteria. Thus enhancing host immunity (20). *Akkermansia* is one of the bacteria that inhibits LPS production and can reduce *endotoxemia*, protect the intestinal barrier, and improve obesity and diabetes (38). *Allobaculum* is one of the bacteria that produce short-chain fatty acids (40). The abundance of Lactobacillus, *Akkermansia,* and *Allobaculum* in the gut decreased significantly after feeding a high-fat diet, while the abundance of these three genera increased significantly after feeding two kinds of seaweed polysaccharides and their oligosaccharides, almost returning to the level of normal diet mice. *Alistipes* metabolizes butyric acid, acetic acid, and propionic acid, which play an important role in anti-inflammatory effects (39). After feeding a high-fat diet, the abundance of *Alistipes* in the intestines of mice was significantly decreased, while after feeding three kinds of seaweed polysaccharides and oligosaccharides, the abundance of *Alistipes* was significantly restored, almost returning to the level of mice with a normal diet. Studies have shown that Helicobacter is a harmful bacterium, and its increased abundance can lead to upper digestive tract diseases (41). After feeding a high-fat diet, the abundance of Helicobacter in the gut of mice increased significantly, which is one of the reasons why a high-fat diet can cause upper digestive tract diseases in mice. However, after feeding two kinds of seaweed polysaccharides and their oligosaccharides, the abundance of Helicobacter decreased significantly, which significantly reduced the risk of upper digestive tract diseases caused by a high-fat diet.

Abnormal lipid metabolism can lead to various diseases. In this study, sodium alginate gel and oligosaccharides were added to the diets of mice with a high-fat diet to investigate the effects of polysaccharides and oligosaccharides derived from seaweed on lipid metabolism and gut microbiota. The results showed that adding seaweed polysaccharides or oligosaccharides to the high-fat diet could inhibit the increase of body weight and regulate blood lipids in mice, and at the same time inhibit the accumulation of fat in the liver of mice, protect the intestinal villus morphology of mice so that it can play a normal function, and play a role in regulating lipid metabolism in mice. The effect of oligosaccharides was better than that of polysaccharides, and the effect of sodium alginate was better than that of agar gel. This study investigated the effects of sodium alginate agalogel and its oligosaccharides on lipid metabolism in high-fat diet mice, and analyzed the changes in gut microbiota abundance related to lipid metabolism in order to provide direction for the prevention and treatment of lipid metabolic diseases, and lay a scientific foundation for the development of marine active substances in marine functional foods.
